# Tympanometry with 226 and 1000 Hertz tone probes in infants

**DOI:** 10.1590/S1808-86942012000100015

**Published:** 2015-10-20

**Authors:** Luciana Macedo de Resende, Juliana dos Santos Ferreira, Sirley Alves da Silva Carvalho, Isamara Simas Oliveira, Iara Barreto Bassi

**Affiliations:** a MSc in Speech and Hearing Therapy - Pontifícia Universidade Católica de São Paulo. (Assistant Professor - Department of Speech and Hearing Therapy Medical School of the Federal University of Minas Gerais - UFMG; PhD Student - Human Communication Disorders at the Federal University of São Paulo - UNIFESP); b Speech and Hearing Therapist - Federal University of Minas Gerais.; c PhD in Sensorial Biophysics - Université D´Auvergne (Adjunct Professor - Department of Speech and Hearing Therapy of the Medical School - UFMG); d MD. ENT Resident - UFMG); e MSc in Health and Occupational Medicine- Graduate Program in Public Health - Department of Social and Preventive Medicine - UFMG. (Speech and Hearing Therapist); PhD student in Health and Occupational Medicine - Graduate Program in Public Health - Department of Social and Preventive Medicine - UFMG). Federal University of Minas Gerais.

**Keywords:** acoustic impedance tests, hearing, infant, infant newborn

## Abstract

This study aimed at describing and analyzing tympanometric results obtained with 226Hz and 1000Hz probe tones; checking for correlations between tympanometry, otoacoustic emissions and otoscopic examination; describing abnormal results found in the evaluation procedures.

**Methods:**

Double-blind and prospective study. Our sample included 70 babies, between 7 days and one month and 13 days of age, without risk indicators for hearing loss, evaluated in the State Neonatal Hearing Screening Program. Transient evoked otoacoustic emissions, otoscopic examination and tympanometry with 226Hz and 1000Hz probe tones were used as assessment tools. The study was approved by the Ethics Committee from the institution.

**Results:**

Statistically significant differences were observed (*p*<0.05) in the tympanometric measures correlation and also between transient evoked otoacoustic emissions and compliance obtained with both probe tones. Most test results were within the normal range (94.28%). Three children (4.28%) were referred to diagnostic follow-up and one (1.42%) had middle ear dysfunction confirmed by otoscopy and 1000Hz tympanometry.

**Conclusions:**

1000Hz tympanometry is the most reliable probe tone used to evaluate children under three months of age. More studies focusing on middle ear acoustics and mechanics are necessary to provide reliable and precise interpretation in the evaluation of middle ear functions in babies.

## INTRODUCTION

Middle ear disorders in newborns and babies have a significant prevalence, and when unidentified, they may delay proper audiological diagnosis. In recent decades, many studies were dedicated to describing the best procedures for a proper assessment at this age range. Most of the studies agree that as far as tympanometry is concerned, the 1000 Hz probe is the most indicated to assess newborns and babies younger than 3 months of age^1^, ^2^, ^3^, ^4^.

Conventional tympanometry is carried out with the 226 Hz probe tone, and the results are reliable for the diagnosis of middle ear disorders in the elderly, adults and in children older than six months of age^5^, ^6^, ^7^.

A study carried out by Linares & Carvallo^8^, with infants between 0 and 8 months of age, advocated the use of the 226 Hz probe in tympanometry, because they found matching results in their assessments, using only this probe tone with the Transient Stimulus Evoked Otoacoustic Emissions (TEOAE), previously carried out in the babies of the sample.

Controversial results regarding the use of the 226 Hz probe tone used only to study tympanometric changes in newborns and babies younger than six months of age have been shown in various studies. At this age range in babies with known middle ear disorders, the tympanograms obtained with the low-frequency test tone probe are not statistically different from those obtained from normal ears. In other words, in infants without TEOAE, there may be a normal tympanometric curve when the 226 Hz probe is used, even in the presence of an air conduction component, which means that the tests carried out with this probe tone have a high rate of false-negative results. Thus, it is considered that such frequency is not very sensitive to detect conduction disorders^9^, ^10^, ^11^, ^12^. In studies carried out by Keefe et al.^13^ and Keefe & Levi^14^, they obtained tympanograms with normal pressure and compliance, coexisting with secretory otitis media, confirmed by the ENT clinical exam. There have also been reported some B-type curve records, according to Jerger's classification^15^, with a normal middle ear.

Kei et al.^1^ carried out a study in which they described the characteristics of low and high frequency in newborns with normal TEOAE results and they used it to establish normative data for the assessment of this population. The 1000 Hz probe tone was considered efficient to be used in NHS (Newborn Hearing Screening), in order to detect possible middle ear disorders in newborns, which may reduce the number of children with audiological diagnosis delayed by chronic otitis media.

The 1000 Hz tone probe was deemed the most adequate to assess newborns and babies younger than 3 months of age in the study carried out by Alaerts et al.^4^. The authors suggested the following protocol: babies younger than 3 months of age must be evaluated solely with a 1000 Hz probe tone; between 3 and 9 months of age a tympanometry must be carried out with the 1000 Hz probe tone; and in cases of failure, a second assessment must ensue with the 226 Hz probe tone only. Another important piece of information present in this study is that the use of the 1000 Hz tone probe reduced the prevalence of flat tympanograms in this group of children.

Thus, the use of this high frequency (1000 Hz) tone test has been suggested by most of the authors, since it has a greater sensitiveness to identify mild middle ear disorders, and it may serve to reduce delays in the diagnosis of conductive hearing loss in children^2^, ^3^, ^16^, ^17^.

The different results found in the studies with the two probe tones (226 Hz and 1000 Hz), in other words, the uncommon characteristics of the tympanograms obtained from newborns and babies, may be attributed to the physiological differences concerning the ears from newborns and adults. During growth, many changes happen in the ears of babies, having impacts on the mechanical properties of the auditory canal, influencing the recording of the tympanograms obtained from newborns and babies, which can be associated with physiological differences between the ears of newborns and adults. During growth, many changes happen to the ears of babies, which impact on the mechanical properties of the auditory canal, influencing the recording of the tympanogram. Among the physical changes seen in the external ear and in the middle ear after growth, which attempt to explain the acoustic changes, including enlargement of the external ear, mastoid and middle ear cavity; changes in tympanic membrane orientation; tympanic annulus fusion; ME mass reduction (because of changes in bone density and mesenchymal loss); bone formation on the EAM wall; ossicular joint compression; and the stapes coming closer to the annular ligament^5^, ^18^, ^19^, ^20^, ^21^.

Newborn tympanometric characteristics are different from those of adults for one single definition of normality to be attributed, in other words, only one type of tympanometric curve and quantitative values. the baby's middle ear is a system dominated by a mass; while in the adult, stiffness predominates.

Mass components are larger in high frequency probes and lower in their low frequency counterparts^1^, ^5^, ^18^. The normal middle ear is primarily dominated by the stiffness of low frequency sounds (226 Hz). In a higher frequency (for instance: 1000 Hz), the relative participation of each anatomical structure is changed and the acoustic admittance measured at the ME inlet becomes more predominated by the mass^16^.

Another difficulty faced by the low frequency tympanometry carried out in newborns and babies younger than six months is associated with its low sensitivity, in other words, the high level of false-negatives, as per previously reported.

Very few tympanometry studies have been led with infants younger than six months of age. Moreover, the details of the middle ear mechanic and acoustic properties of neonates have not been broadly explored. Both systematic studies as well as normative data are needed in order to enhance the auditory diagnosis in newborns and babies. Studies which contribute to a more in-depth and accurate study on the prevalence of middle ear disorders in babies need to be carried out.

The present study aimed at analyzing the tympanometric results from newborns and babies up to two months of age, obtained in tests with the 226 Hz and 1000 Hz probes; describing and comparing the parameters obtained upon testing (external auditory meatus volume, compliance and tympanometric peak pressure); correlating the sample's tympanometric findings with the TEOAE test results; correlating the otoscopic findings with the tympanometric findings; and describing the tests which showed failures in the sample babies.

## MATERIALS AND METHODS

This was a double-blind prospective study, which sample was made up of 70 newborns and babies, of both genders, with ages between 7 days and one month and 13 days of life.

The newborns and babies included in the study did not have risk indicators for hearing loss according to the criteria proposed by the *Joint Committee on Infant Hearing - JCIH*^22^ and the recommendations of the Brazilian Committee on Hearing Loss in Childhood (CBPAI)^23^, born at term and who did not have a background of pre, upon birth and postnatal complications which showed some hearing change.

The parents or guardians of the children were explained about the aims of the study and the procedures to be carried out. It was stressed that they were non-invasive, painless and did not carry risks for those participating in the study. All the parents agreed with and authorized the use of the results from the tests in the present study after signing the informed consent form.

The sample's exclusion criteria were: any type of external auditory canal malformation, neurological changes and/or some type of genetic syndrome, and the parents/guardians disagreeing with signing the informed consent.

After carrying out the Universal Neonatal Hearing Screening, made up by the Transient Evoked Otoacoustic Emissions and Observation of the auditory behavior; the newborns and babies were submitted to otoscopic examination, done by an otorhinolaryngologist. Immediately afterwards, the tympanometric test was carried out with the 226 Hz and 1000 Hz probe tones.

The data collected in each evaluation was recorded in protocols set up for this specific study. The examiners, as well as those responsible for the babies were not told about the results from the tests of the other babies. The researcher only had access to the results after the end of the tests to which the babies were submitted to. The parents were told about the evaluation results after the end of all the tests.

The Otoacoustic Emissions test is a non-invasive, fast and objective test, sensitive to detect unilateral or bilateral, mild to profound hearing loss, and it can be done in places without sound proof facilities. Transient Evoked Otoacoustic Emissions were carried out with non-linear clicks at 80 dBSPL and the test window was of 12 milliseconds, with 512 stimuli (screening protocol utilized). The emissions were recorded using an AuDX Plus device, from Biologic Co. (USA). In the test, the parameters analyzed were: the presence and reproducibility of the emissions and the signal/noise ratio.

Otoscopy is the visual inspection of the external acoustic meatus and the tympanic membrane. The otorhinolaryngologist is the physician who performs the test with an otoscope, introducing a small speculum in the baby's ear and shedding light in the external auditory canal all the way to the tympanic membrane. It is a common procedure, aimed at diagnosing different disorders of the external auditory canal, such as inflammation, infections, foreign bodies, wax, and others. It is also useful in order to detect changes in the tympanic membrane, which may be translated as opacity, hyperemia, bulging, perforation and retraction.

Tympanometry is a dynamic and objective test which provides information about the compliance or mobility of the tympanum-ossicular system in function of changes to the air pressure in the external auditory canal. One probe was introduced in the external auditory canal of the baby, by means of which a variable pressure of +200 daPa to −400 daPa was employed, with a speed of 50 daPa/s. All the tests were carried out with the child naturally sleeping in the arms of the mother or caretaker, to have the child as calm as possible. The babies were submitted to the test without a strict order of presentation of the test tone. However, most of the tests started at the frequency of 226 Hz followed by the frequency of 1000 Hz. The choice of ear to start the test was random, which depended on the positioning of the baby in the arms of the mother or caretaker. When the tympanometric curve was not satisfactorily obtained, because of baby movement, causing pressure escape, the test was repeated, removing the probe and re-inserting it in the same ear in order to obtain new reliable values.

After the end of all the procedures, the tests were analyzed and normality criteria were established:

- The Transient Evoked Otoacoustic Emissions were considered present when the reproducibility was higher than or equal to 70%, the minimum intensity of emission recording (emission amplitude) was equal to or higher than 6dBSPL, the signal/noise ratio (TE/ NF) was equal to or higher than 6dBSPL and the probe stability was kept at 90% or higher. Then, the otoacoustic emissions were classified as Present or Absent. The recording of the responses carried out by means of an AuDX Plus, from Biologic, with the *Scout Sport (USA)* used to analyze the responses.
- Otoscopic findings were described as normal or changed; and the changes signs listed were: opacity, hyperemia, bulging, perforation and retraction of the tympanic membrane.
- The tympanometric test was carried out by the researcher using two probe tones, 226 Hz and 1000 Hz. The recording was made with the *Impedance Audiometer AT 235h* middle ear analyzer, by *Interacoustics,* the automatic version, calibrated according to the manufacturer's instructions (Assens, Denmark). The parameters observed during the test carried out with the two probe tones were described and analyzed, namely: Tympanometric Peak Pressure, External Ear Volume and Compliance.

In the statistical study, we carried out descriptive and comparative analyses and found an association between the results from the Neonatal Auditory Screening, otoscopy and tympanometry of the neonates and babies. the analyses were carried out by the Wilcoxon test and the mean values were calculated. For statistical inference analyses purposes, a 0.05 significance level was defined and the significant values were marked by an asterisk (*).

The present study, as well as the Informed Consent Form were approved by the Ethics in Research Committee of the Institution on September 02, 2009, under document # ETIC 337/09.

## RESULTS

The results were presented by ear (right and left) and by test carried out. We carried out a descriptive analysis of the tests which were changed in the sample babies. We recorded 140 tympanograms for each test tone frequency, 226 Hz and 1000 Hz.

In the statistical analyses of the data, we made the following correlations: comparison between the parameters of the tests carried out with the probe tones of 226 Hz and 1000 Hz, aiming at checking for a statistically significant difference; a correlation between the TEOAE results and the compliance values obtained with the different probe tones.

The results from Tables 1 and 2 pointed to a statistical difference in comparing the two probe tones, in each ear, according with the tympanometric measures analyzed: External Ear Volume, Tympanometric Peak Pressure (TPP) and Compliance.

**Table 1:** Tympanometry values measures with the 226 Hz and the 1000 Hz probes in the RE.

	Vol. EE		TPP (daPa)		Compliance	
Tymp RE	226 Hz (ml)	1000 Hz (mmho)	226 Hz	1000 Hz	226 Hz (ml)	1000 Hz (mmho)
Mean	0.64	0.42	1.39	19.68	0.91	0.72
St. Deviation	0.15	0.08	40.92	45.30	0.48	0.37
*p* Value	0.000*		0.005*		0.010*	

Tymp: tympanometry; RE: right ear; Vol. EE: external ear volume;

TPP: tympanometric peak pressure.

* Wilcoxon test *p* < 0.05.

**Table 2:** Tympanometry values with the 226 Hz and 1000 Hz probe in the RE.

	Vol. EE		TPP (daPa)		Compliance	
Tymp LE	226 Hz (ml)	1000 Hz (mmho)	226 Hz	1000 Hz	226 Hz (ml)	1000 Hz (mmho)
Mean	0.64	0.43	3.03	16.05	0.98	0.79
Std. Deviation	0.14	0.16	40.44	48.48	0.51	0.48
*p* Value	0.000*		0.078*		0.009*	

Tymp: tympanometry; RE: right ear; Vol. EE: external ear volume;

TPP: tympanometric peak pressure

* Wilcoxon test *p* < 0.05.

The correlation between the TEOAE results and the compliance values obtained with the different probe tones also proved to be statistically significant in both ears, according to Tables 3 and 4.

**Table 3:** Comparison between TEOAE amplitude and compliance at 226 Hz and 1000 Hz in the RE.

	Amplitude RE(dBSPL)	Compliance	
		226 Hz (ml)	1000 Hz (mmho)
Mean	19.70	0.91	0.72
Standard Deviation	5.28	0.48	0.37
*p* Value		0.000*	0.000*

RE: right ear

* Wilcoxon test *p* < 0.05.

**Table 4:** Comparison between TEOAE amplitude and compliance at 226 Hz and 1000 Hz in the RE.

	LE Amplitude (dBSPL)	Compliance	
		226 Hz (ml)	1000 Hz (mmho)
Mean	17.95	0.98	0.79
Standard deviation	5.69	0.51	0.48
*p* Value		0.000*	0.000*

LE: left ear

* Wilcoxon test *p* < 0.05.

As far as the descriptive analysis is concerned, we noticed that of the 70 babies assessed, 66 (94.28%) had normal results in all the tests. Only four had changed results in one or more of the tests carried out. Of these, two babies did not have TEOAE, in other words, failed the Neonatal Auditory Screening. Upon the tympanometric test carried out with two probe tones, the results obtained were normal and upon otoscopic evaluation we also did not see tympanic membrane abnormalities. Thus, the babies did not have any middle ear changes which would justify the lack of the emissions. These babies were referred to audiological diagnosis.

Only one baby failed auditory screening and the tympanic membrane was opaque upon otoscopic evaluation. The middle ear disorder was confirmed by the tympanometry done with the 1000 Hz tone probe, in which we found a B-type curve, according to the classification by Jerger^18^, which matches the lack of TEOAE. On the other hand, upon the tympanometric test carried out with the 226 Hz tone probe, there was a type-A false curve.

Only one baby, who failed auditory screening, had changes in the tympanic membrane (opacity) upon otoscopy and, on the other hand, we obtained normal results upon the tympanometric test with the two probe tones. These findings make up 1.42% of the hearing disorders found in the sample. Only three patients in the sample (4.28%) required referral to a hearing diagnosis with suspicion of sensorineural hearing loss.

## DISCUSSION

The main goal of the present study was to study the relationship between the TEOAE, the otoscopic findings and the tympanometric measures obtained with the 226 Hz and 1000 Hz probes in newborns and babies, in an attempt to obtain information which help in the diagnostic decision, based on the evaluation carried out by these tests. Few tympanometric studies with newborns and babies younger than six months of age have been carried out and are necessary to improve the use of tympanometric responses in the auditory diagnosis at this age range.

The greater difficulty found by researches is standardizing tympanometry in infants, since, having no Otoacoustic Emissions, during newborn hearing screening, the concern is with the differentiation between the involvement of the middle ear and internal ears^8^.

In the present study, after analyzing the quantitative measures recorded in the tympanometry carried out with the 226 Hz and 1000 Hz, probe tones, the mean values found were: for the right ear - the external ear volume was 0.64ml with the 226 Hz tone and 0.42mmho with 1000 Hz; the TPP varied between −96daPa and 93daPa, with a mean value of 1.39daPa for the 226 Hz tone and between −129daPa and 100daPa, with a mean value of 19.68daPa for the 1000 Hz test tone; the compliance values were 0.91ml with the 226 Hz test tone and 0.72mmho with 1000 Hz. For the left side - the external ear volume was 0.64ml, with the 226 Hz tone and 0.44mmho with 1000 Hz; TPP varied between −87daPa and 72daPa, with a mean value of 3.03daPa for the 226 Hz tone and between −119daPa and 100daPa, with a mean value of 16.05daPa for the 1000 Hz tone test; compliance values were 0.98ml with the 226 Hz tone test and 0.79mmho with the 1000 Hz tone. The comparison of the mean values obtained from both tone probes was statistically significant, which is translated into the difference in the quantitative values when tympanometry is carried out with the different probe tones.

These results are similar to the ones reported by Carvallo^24^ in relation to the quantitative measures, the mean values found were: external ear volume of 0.55ml with the 226 Hz tone, 1.29mmho with 678 Hz and 1.67mmho with 1000 Hz; compliance of 0.56ml with the 226 Hz test tone, 0.45mmho with 678Hz and 0.84mmho with a 1000Hz test tone; TPP varied between −95daPa and 60daPa with the 226Hz tone, between −120daPa and 120daPa with the 678Hz tone and between −150daPa and 115 daPa with the 1000Hz test tone.

In the study carried out by Kei et al.^1^, 170 healthy newborns were assessed by means of the TEOAE and tympanometry with the 226 Hz test tone and 1000 Hz. We noticed an ear significance effect, in which the right ears had higher mean static admittance values; however, acoustic admittance lower mean values at +200 daPa, compared to the left ears. We did not find a significance effect associated to gender or interactions between the ears.

On the other hand, in a study carried out by Alaerts et al.^4^ they reported that in infants younger than 3 months of age, tympanograms with test tone of 1000 Hz were easier to interpret and had more reliable values (91%) than the 226 Hz (35%) tone, which shows a significantly better result in the assessment of the middle ear system. Moreover, the 226 Hz test tone resulted in 58% of false-positive results in this age range. the external ear volume and compliance values obtained by the authors are different from those found in the present study.

Indications for using the 1000 Hz test tone in the tympanometry of babies come from middle ear physiological and anatomical differences, as per described by Holte et al.^18^, Hall & Chandler^19^, Keefe et al.^20^, Margolis & Hunter^5^. Such occurrences are expected, because the external and middle ear structures of newborns and babies change as they grow and develop, becoming as the adult ears at about nine years of age^20^.

Studies carried out by Margolis et al.^2^ and Kei et al.^1^ alluded to evidence favoring the 1000 Hz test tone probe when compared to the 226 Hz and 678 Hz probes, since they noticed that the mass components are higher in the high-frequency probes and lower in the low low-frequency probes, which change the middle ear resonance characteristics. Such statements help in the justifications of the findings of this study.

Another goal of the present study was to check the relationship between the multiple frequency tympanometry and the TEOAE in order to analyze which probe tone had the best correlation. In our sample, we found statistically significant differences in the correlation of the TEOAE amplitude with the tympanometry test carried out with the two probe tones for both ears. As per depicted on Tables 3 and 4, the mean values recorded for TEOAE amplitude values and compliance in 226 Hz and 1000 Hz in the right ear were, respectively: 19.70dBSPL, 0.91ml and 0.72mmho; and for the left ear we found:17.95dBSPL for TEOAE amplitude, 0.98ml and 0.79mmho for compliance in 226 Hz and 1000 Hz, respectively.

In the study carried out by Garcia et al.^7^, concerning the comparison between the tympanometric test - using the 226 Hz probe tone and the presence or absence of TEOAE in the two groups studied, they did not find any statistically significant difference. On the other hand, the 1000 Hz test tone yielded a statistically significant correlation in the two groups. Group I (TEOAE present) was normal in 81.8% of the tympanometries and in Group II (absent TEOAE) 88.9% with tympanometric changes.

The same occurrence was also noticed in numerous studies, in which the authors found a significant TEOAE correlation with the tympanometric exam - only when carried out with the 1000 Hz test tone in newborns and babies^2^, ^3^, ^17^.

We noticed a major variability of responses comparing the findings of the present study with the literature, especially the TEOAE amplitude parameters and tympanometry compliance; which is due to numerous factors, among them the size of the sample considered, testing conditions, whether or not there was baby movement or sucking at the time of recording, proper sealing of the external auditory meatus, gestational age, ear size and the baby's general health.

It is worth noting that in this study we found a lower prevalence of conduction disorders (1.42%), proven by otoscopy and tympanometry with the 1000 hz probe tone. Such result contradicts the study carried out by Linares & Carvallo^8^, in which the group without TEOAE showed 33.3% of type-B curve tympanograms. In the study carried out by Carmo^17^, the study group, without TEOAE, had 74.07% of changed tympanograms, when analyzed by the 1000 hz test tone probe. the same happened in the study carried out by Garcia et al.^7^, in which 95% of the ears assessed in the study group (infants without TEOAE and changes upon otoscopic evaluation) were altered upon the tympanometric test done with the 1000 Hz. Contrary to what was found in the assessment using the 226 Hz probe, 88.9% of the tympanograms were normal, even when changes were found upon otoscopy. Such differences between the studies are probably due to sample size and the health situation of the newborns and babies at the time of the test.

Studies show different prevalence of normal tympanograms, with the 1000 Hz probe, in babies up to three months of age, regardless of the classification utilized. Such differences are due to the number of babies included in each study. Margolis et al.^2^ found 91% of normal results and Baldwin^25^ found 71% of normal tympanograms. Kei et al.^1^ found 92.2% of tympanograms with a single peak, similar to type A, which indicates normal middle ear function; 5.7% flat-curve tympanograms, similarly to type B; 1.2% had double peak tympanograms and other atypical forms happened to both ears (0.8%) of the sample studied. Such results corroborate those of the present study, which found 94.28% of normal results with the 1000 Hz test tone.

Tympanometry, especially the one carried out with the high frequency test tone, may help in the auditory diagnosis of newborns and babies up to six months of age. However, it must not be interpreted alone, but together with the results from the other tests - TEOAE and otoscopy.

There are very few studies in the literature considering tympanometry in newborns and babies. There is a need to do more studies, so as to have more data to be compared and, thus, conferring greater reliability to the interpretation of the tests and validation of the results in patients at this age range.

## CONCLUSIONS

The parameters obtained and analyzed in the correlation between the tympanometric tests performed with the 226 Hz and 1000 Hz probe tones have statistically significant differences. The correlation between TEOAE results and the compliance values also proved to be statistically different vis-à-vis the different probe tones. Most babies assessed had normal results in all tests performed. There was only one baby who failed auditory screening and his middle ear disorder was proven statistically significant with the different probe tones. Most of the babies assessed had normal tests. Only one baby failed hearing screening and the middle ear change was seen upon otoscopy and the tympanometry carried out with the 1000 Hz. Results indicated that the 1000 Hz probe tone is the one most recommended for the assessment of babies up to 3 months of age.

## References

[1] Kei J, Allison-Levick J, Dockray J, Harrys R, Kirkegard C, Wong J (2003). High-frequency (1000 Hz) tympanometry in normal neonates. J Am Acad Audiol..

[2] Margolis RH, Bass-Ringdahl S, Hanks WD, Holte L, Zapala DA (2003). Tympanometry in newborn infants - 1kHz norms. J Am Acad Audiol..

[3] Calandruccio L, Fitzgerald TS, Prieve BA (2006). Normative multifrequency tympanometry in infants and toddlers. J Am Acad Audiol..

[4] Alaerts J, Luts H, Wouters J (2007). Evaluation of middle ear function in young children: clinical guidelines for the use of 226-and 1,000-Hz timpanometry. Otol Neurotol..

[5] Margolis RH, Hunter LL, Musiek FE, Rintelmann WF (2001). Perspectivas Atuais em Avaliação Auditiva.

[6] Carvallo RMM, Carvallo RMM (2003). Fonoaudiologia: informação para formação.

[7] Garcia MV, Azevedo MF (2009). Testa, JR. Accoustic immitance measures in infants with 226 and 1000 hz probes: correlation with otoacoustic emissions and otoscopy examination. Braz J Otorhinolaryngol..

[8] Linares AE, Carvallo RMM (2008). Acoustic immittance in children without otoacoustic emissions. Braz J Othorinolaryngol..

[9] Paradise JL, Smith CG, Bluestone CD (1976). Tympanometric detection of middle ear effusion in infants and young children. Pediatrics..

[10] Shurin PA, Pelton SI, Klein JO (1976). Otitis media in the newborn infant. Ann Otol Rhinol Laryngol..

[11] Hunter LL, Margolis RH (1992). Multifrequency tympanometry: current clinical application. Am J Audiol..

[12] Meyer SE, Jardine CA, Deverson W (1997). Developmental changes in tympanometry: a case study. Br J Audiol..

[13] Keefe DH, Bulen JC, Arehart KH, Burns EM (1993). Ear-canal impedance and reflection coefficient in human infants and adults. J Acoust Soc Am..

[14] Keefe DH, Levi E (1996). Maturation of the middle and external ears: acoustic power-based responses and reflectance tympanometry. Ear Hear..

[15] Jerger J (1970). Clinical experience with impedance audiometry. Arch Otolaryngol..

[16] Silva KAL, Novaes BACC, Lewis DR, Carvallo RMM (2007). Tympanometry in neonates with normal otoacoustic emissions: measurements and interpretation. Braz J Otorhinolaringol..

[17] Carmo MP (2009). Imitanciometria com sonda de baixa e alta frequ-ência em lactentes com indicadores de risco para a deficiência auditiva [Tese].

[18] Holte L, Margolis RH (1991). Cavanaugh RM Jr. Developmental changes in multifrequency tympanograms. Audiology..

[19] Hall III JW, Chandler D, Katz J. (1999). Tratado de Audiologia Clínica.

[20] Keefe DH, Folsom RC, Gorga MP, Vohr BR, Bulen JC, Norton SJ (2000). Identification of neonatal hearing impairment: ear-canal measurements of acoustic admittance and reflectance in neonates. Ear Hear..

[21] Northern JL, Downs MP (2005). Audição na Infância.

[22] American Academy of Pediatrics, Joint Committee on Infant Hearing (2007). Year 2007 position statement: principles and guidelines for early hearing detection and intervention programs. Pediatrics..

[23] Comitê Brasileiro sobre Perdas Auditivas na Infância (CBPAI) (2000). Recomendações 01/99 do Comitê Brasileiro Sobre Perdas Auditivas na Infância. J Cons Fed Fonoaudiol..

[24] Carvallo RMM (1992). Medida de imitância acústica em crianças de zero a oito meses de idade [Tese]..

[25] Baldwin M (2006). Choice of probe tone and classification of trace patterns in tympanometry undertaken in early infancy. Int J Audiol..

